# Clinical Approach and Successful Intervention of Congenital Hydrocephalus in Neonatal Calf

**DOI:** 10.1002/vms3.70657

**Published:** 2025-10-14

**Authors:** Antora Akter, Amir Hamza Masum, Rezwanul Huq, Mahmudul Alam

**Affiliations:** ^1^ Department of Surgery and Obstetrics Faculty of Veterinary Science Bangladesh Agricultural University Mymensingh Bangladesh

**Keywords:** calf, hydrocephalus, recovery, surgery

## Abstract

Congenital hydrocephalus is a potentially fatal neurological anomaly in calves characterized by excessive accumulation of cerebrospinal fluid that leads to cranial deformities and neurological deficits. This report describes the successful surgical management of a day‐old crossbred female calf presenting with a dome‐shaped cranial vault, poor suckling reflex and uncoordinated gait. Diagnosis of congenital hydrocephalus was confirmed by clinical examination. Surgical intervention involved gradual aspiration of cerebrospinal fluid using a syringe, followed by excision of the enlarged area at the level of a horizontal mattress suture placed at the base of the swelling. Post‐operative care included antibiotics, corticosteroids and fluid therapy. Progressive neurological improvement was noted within 72 h, and by 12 days post‐surgery, the calf exhibited normal growth, behaviour and no signs of neurological dysfunction. This report represents a successful outcome following surgical management of congenital hydrocephalus in a neonatal calf.

## Graphical abstract

A neonatal calf with congenital hydrocephalus, characterized by a dome‐shaped cranial vault, was treated with gradual drainage of cerebrospinal fluid and a horizontal mattress suture. The calf exhibited neurological improvement following surgery. At 12‐day follow‐up, normal behaviour and growth were observed with no neurological deficits.

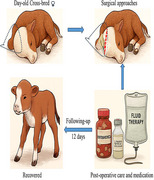

## 1 Introduction

Congenital hydrocephalus is a neurologically debilitating condition characterized by abnormal accumulation of cerebrospinal fluid (CSF) within the ventricular system of the brain, leading to increased intracranial pressure, cortical atrophy and neurobehavioural deficits (Kahrs et al. 1970; Leech et al. 1978). In neonatal calves, this condition, although rare, poses significant clinical and diagnostic challenges due to its non‐specific early signs and the limitations in diagnostic resources in large animal practice (Leipold and Dennis 1987). The aetiology of congenital hydrocephalus in calves is multifactorial, often involving genetic predisposition, teratogenic exposure, or in utero viral infections such as bovine viral diarrhoea virus (BVDV) or bluetongue virus that have been implicated in disrupting foetal neurodevelopment, resulting in ventricular dilatation, cerebral hypoplasia and other CNS malformations (Scott et al. 1973; Constable et al. 2017; Newcomer et al. 2012). Clinical manifestations of congenital hydrocephalus typically include an enlarged, dome‐shaped skull, uncoordinated movement, blindness and ataxia. While these signs indicate potential neurological dysfunction, they may not fully reflect the extent of the condition. Therefore, a thorough neurological evaluation, including assessment of cranial nerve function, postural reflexes and gait, is essential to confirm the diagnosis and determine the severity of neurological impairment. Without timely and appropriate intervention, affected neonates often fail to thrive and may eventually succumb to complications arising from increased intracranial pressure (Leech et al. 1978; De Lahunta and Glass 2009; Dhami et al. 2007).

In veterinary practice, diagnosis primarily relies on a combination of clinical presentation and imaging modalities such as transcranial ultrasonography and radiography, which provide a non‐invasive assessment of ventricular dilatation (Gorjidooz et al. 2021; Neogy et al. 2014). However, treatment of congenital hydrocephalus in food animals is infrequent and mostly palliative. Surgical interventions such as ventriculoperitoneal (VP) shunting are standard in small animal practice but seldom reported in large animal practice due to technical complexity, cost constraints and lack of specialized infrastructure (Platt and Garosi 2012; Shihab et al. 2011). However, emerging evidence suggests that shunting can be a viable and successful option in selected large animal cases when performed under appropriate surgical and post‐operative protocols (Long 2001).

This case report presents a rare and successful case of congenital hydrocephalus in a neonatal calf managed through surgical intervention, and this can be feasible and effective in large animals under field conditions. To our knowledge, this is one of the few documented cases reporting favourable outcomes following surgical intervention for hydrocephalus in bovine neonates.

## 2 Case History and Clinical Assessment

**FIGURE 1:**
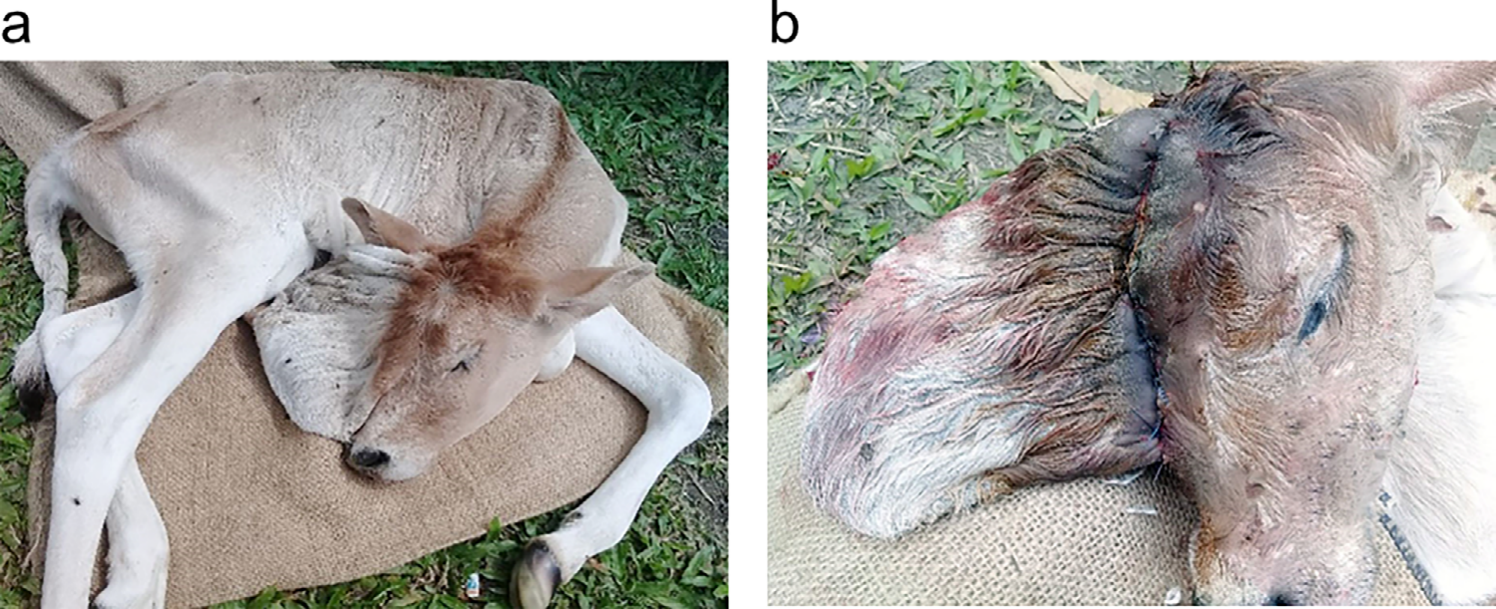
Calf with congenital hydrocephalus following surgical intervention. (a) Congenital hydrocephalus of calf. (b) Reduction in head size after drainage of cerebrospinal fluid and horizontal mattress suture was placed at the base of the swelling to minimize dead space.

A day‐old female crossbred calf was presented to the Veterinary Teaching Hospital at Bangladesh Agricultural University in Mymensingh, with complaint of abnormal cranial morphology, dome‐shaped skull (the frontal skull bones in that area were not developed, and a hollow filled with fluid was found beneath the skin), fatigue, a poor latching reflex, head twitches, convulsions and lying in a lateral recumbent position (Figure 1a). The calf was born full‐term via normal parturition, and no similar congenital defects had been observed in the herd's history. The dam received standard vaccinations, and there were no known teratogenic exposures during gestation.

On physical examination, the calf was dull but responsive and remained in lateral recumbency, unable to rise without assistance. Neurological examination revealed a mild‐to‐moderate head tilt with ventrolateral strabismus on the left side. Cranial nerve evaluation showed reduced menace response bilaterally, normal pupillary light reflexes and intact palpebral reflexes. No abnormal nystagmus was observed. The calf exhibited intermittent opisthotonos and paddling movements consistent with seizure‐like activity during the examination. Proprioceptive reflexes could not be adequately assessed due to the recumbent condition. These findings indicated marked central nervous system dysfunction consisting of congenital hydrocephalus. From case history and clinical examination, it was determined that the calf would be operated on and given medical attention with the owner's approval.

## 3 Surgical Management

Animal received 200 mL of mannitol (20%) intravenously at the dose rate of 0.5 g/kg BW as preoperative treatment to reduce intracranial pressure. The area at the swelling base was then meticulously cleaned and shaved. For local analgesia, 2% lidocaine hydrochloride (Lidocaine Plus, Techno Drugs Ltd., Narsingdi, Bangladesh) at the dose rate of 4 mg/kg BW was injected subcutaneously around the swelling area of the head. After painting the area with povidone‐iodine, a horizontal mattress suture made of nylon suture material was placed at the base of the swelling after the fluid was progressively drained out using a syringe (Figure 1b). Following this, the redundant skin and connective tissue forming the dome‐shaped protrusion were excised surgically at the base of the swelling, where a horizontal mattress suture was placed to ensure closure and minimize dead space.

## 4 Post‐Operative Follow‐Up

The calf was given a 400 mL slow intravenous infusion of 25% Dextrose in Normal Saline (DNS) at a dose of 1 mL/kg BW, as well as the antibiotic ceftriaxone at a dose of 25 mg/kg BW was injected intramuscularly for 7 days and corticosteroid at a dose of 1 mg/kg BW was injected intramuscularly for 3 days, and an antiseptic suture line dressing with povidone iodine every day for 10 days after the procedure. Following the surgery, the calf began suckling within 10 h, indicating an early return of neurological function. The surgical site healed without complications, and the sutures were removed on the 12th post‐operative day. No signs of infection or inflammation were observed during the healing period. According to the owner's report, the calf remained clinically healthy and behaviourally normal for 1 month following the surgery, with no recurrence of neurological signs.

## 5 Discussion

Congenital hydrocephalus results from an imbalance between CSF production and absorption, often due to obstruction of flow or impaired reabsorption (Constable et al. 2017; Newcomer et al. 2012), resulting in increased intracranial pressure, cerebral compression and variable neurological dysfunction (Sharma et al. 2015). This case describes a day‐old female crossbred calf presenting with classical signs, including a dome‐shaped skull, convulsions, reduced suckling reflex and lateral recumbency, all of which are hallmark indicators of elevated intracranial pressure and cortical impairment (Leipold and Dennis 1987; De Lahunta and Glass 2009). The physical finding of undeveloped frontal bones and a fluid‐filled cavity beneath the scalp is suggestive of severe ventriculomegaly or hydranencephaly, and likely points to either a non‐communicating hydrocephalus or an extreme form of cortical dysgenesis (Abubakr et al. 1998; Sharda and Ingole 2002; Neogy et al. 2014). Dome‐shaped cranial vaults and persistent open fontanelles have been well‐documented as visible indicators of hydrocephalus in both bovine and small animal neonates (Dhami et al. 2007; Whitlock et al. 2008; Constable et al. 2017). In this calf, these features, coupled with seizures and poor suckling, strongly supported a neurological basis for the presentation. In this case, there was no herd history of similar congenital defects, the dam was appropriately vaccinated and no known teratogenic exposure occurred during gestation. This makes sporadic congenital malformation or an unrecognized genetic mutation more likely. Similar cases have also been documented in Holstein and crossbred calves without identifiable infectious causes, supporting the possibility of idiopathic or hereditary aetiologies (Dhami et al. 2007).

Previous reports have mostly recommended euthanasia or palliative care for neonatal hydrocephalus due to poor prognosis and lack of practical therapeutic options in production animals (Dore and Smith 2017; Gorjidooz et al. 2021). However, in this case, with the owner's approval, a surgical approach was attempted to manage the condition. This decision was based on the severity of neurological signs and the potential to improve the calf's quality of life with timely intervention. It reflects an important shift in veterinary thinking, where the clinical value of life‐saving surgical intervention in livestock is weighed not only against economic utility but also against ethical and welfare considerations.

Surgical intervention in calves with congenital hydrocephalus provided temporary relief and allowed some animals to survive for several days to weeks, particularly when appropriate post‐operative management was ensured. Nevertheless, complete recovery was not achieved in severe cases, where neurological deficits persisted. These findings suggest that the prognosis of hydrocephalus following surgical treatment is variable and largely dependent on both the severity of the malformation at presentation and the quality of post‐operative care.

The limitations of this case include the lack of pre‐operative advanced imaging such as x‐ray, ultrasonography or magnetic resonance imaging, which would have provided more definitive anatomical detail prior to surgery. However, such imaging is often unavailable in veterinary field settings in developing countries.

## 6 Conclusion

This case contributes to the limited body of literature describing interventional approaches to congenital hydrocephalus in neonatal ruminants, particularly under field conditions. It demonstrates that, with early recognition and supportive surgical management, there may be opportunities to prolong life and improve neurological function, even in cases previously thought untreatable.

## Author Contributions

**Mst. Antora Akter**: conceptualization, methodology, supervision, writing – review and editing. **Amir Hamza Masum**: methodology, writing – original draft. **Md. Rezwanul Huq**: methodology, writing – original draft. **Md. Mahmudul Alam**: methodology, supervision, writing – review and editing. All the authors contributed significantly and have sincerely seen and approved the case report for submission.

## Conflicts of Interest

The authors declare no conflicts of interest.

## Peer Review

The peer review history for this article is available at https://www.webofscience.com/api/gateway/wos/peer‐review/10.1002/vms3.70657.

## Data Availability

All the data on this case report will be available from the corresponding author.
